# Pulmonary artery denervation improves pulmonary arterial hypertension induced right ventricular dysfunction by modulating the local renin-angiotensin-aldosterone system

**DOI:** 10.1186/s12872-016-0366-4

**Published:** 2016-10-10

**Authors:** Chen Liu, Xiao-Min Jiang, Juan Zhang, Bing Li, Jing Li, Du-Jiang Xie, Zuo-Ying Hu

**Affiliations:** 1 Department of Cardiology, Nanjing First Hospital, Nanjing Medical University, 68# Changle Road, Nanjing, 210006 China; 2 Division of Cardiology, Nanjing First Hospital, 68# Changle Road, Nanjing, 210006 China

**Keywords:** Pulmonary artery denervation, Pulmonary arterial hypertension, Right ventricular dysfunction, Renin–angiotensin–aldosterone system

## Abstract

**Background:**

Pulmonary arterial hypertension (PAH) is commonly accompanied with the activation of the renin-angiotensin-aldosterone system (RAAS). Renal sympathetic denervation (RSD) reduces PAH partly through the inhibition of RAAS. Analogically, we hypothesized that pulmonary artery denervation (PADN) could reverse PAH and PAH-induced right ventricular (RV) dysfunction by downregulating the local RAAS activity.

**Methods:**

Twenty-five beagle dogs were randomized into two groups: control group (intra-atrial injection of N-dimethylacetamide, 3 mg/kg, *n* = 6) and test group (intra-atrial injection of dehydrogenized-monocrotaline, 3 mg/kg, *n* = 19). Eight weeks later, dogs in the test group with mean pulmonary arterial pressure (mPAP) ≥25 mmHg (*n* = 16) were reassigned into the sham (*n* = 8) and PADN groups (*n* = 8) by chance. After another 6 weeks, the hemodynamics, pulmonary tissue morphology and the local RAAS expression in lung and right heart tissue were measured.

**Results:**

PADN reduced the mPAP (25.94 ± 3.67 mmHg vs 33.72 ± 5.76 mmHg, *P* < 0.05) and the percentage of medial wall thickness (%MWT) (31.0 ± 2.6 % vs 37.9 ± 2.8 %, *P* < 0.05) compared with the sham group. PADN attenuated RV dysfunction, marked with reduced atrial natriuretic peptide (ANP), brain natriuretic peptide (BNP) and ratio of right ventricular to left ventricular plus septum weight [RV/(LV + S)]. Moreover, the local RAAS expression was activated in PAH dogs while inhibited after PADN.

**Conclusions:**

PADN improves hemodynamics and relieves RV dysfunction in dogs with PAH, which can be associated with the downregulating RAAS activity in local tissue.

## Background

Pulmonary arterial hypertension (PAH) is a lethal disease with poor long-term prognosis and high mortality, which is defined by a mean pulmonary arterial pressure (mPAP) ≥25 mmHg at rest [1]. Recent clinical trials study groups have reported that the mortality rate of French Registry patients at 3–5 years is approximately 20–30 % and the mortality rate of REVEAL Registry patients at 1–3 years is in the range of 10–30 % [2]. The disease is characterized by excessive pulmonary vascular remodeling, contributing to increased pulmonary vascular resistance (PVR) and pulmonary pressure [3, 4]. Increased pulmonary vascular afterload can result in RV adaptation, ultimately leading to RV failure and even death. Besides pressure overload, other factors, such as oxidative stress, ischemia, inflammation, and neurohormonal activation, are also involved in RV remodeling [5]. Actually, the RV function is a major determinant of prognosis in PAH patients [6, 7].

Despite the complex and multifactorial pathogenesis of PAH, neurohormonal activation has been considered as an important factor in the pathophysiology of PAH. Two key players of the neurohormonal system are the sympathetic nervous system (SNS) and the RAAS. It is well established that sympathetic nerve (SN) activity is increased in patients with PAH [8, 9], indicating that hyperactivity of the SNS plays a central role in PAH. Additionally, the RAAS has been proved to be activated when PAH occurs both in animal models and clinically [10–12]. For instance, the increased expression of angiotensin converting enzyme (ACE) along with angiotensin II (Ang II) was observed in the development of pulmonary hypertension (PH) [10, 11]. Elevation of Ang II type 1 receptor (AT1 receptor) was also demonstrated in patients with idiopathic pulmonary arterial hypertension [12]. All the evidence above has illustrated that SNS and RAAS are closely involved in the development of PAH, and may have long-term effects on the progression of PAH.

Pulmonary artery denervation (PADN) is one of the newest potential therapies of PAH. Performed at the bifurcation area of the main pulmonary artery (PA), the ostial right PA and the ostial left PA, by inducing local injury or destruction to the baroreceptor or sympathetic nervous fibers, PADN reduces the pulmonary arterial pressure [13]. It has been demonstrated that PADN conducted in the main pulmonary artery bifurcation area induces severe SN injury and abolishes PAH induced by balloon inflations in a dog model [14]. Reduction of PAH and improvement of right heart function have also been proved clinically in patients with PAH [13]. However, the associated mechanisms of PADN remain unclear. Previous studies have shown that renal sympathetic denervation (RSD) decreases PAP and RV pressure, mediating mainly by inhibiting the activity of the RAAS [15, 16]. Based on the aforementioned knowledge, we hypothesized that PADN may reduce PAH and improve RV function by modulating the local RAAS activity.

In our study, we sought to investigate whether PADN can attenuate PAH and PAH-induced RV dysfunction through downregulating the local RAAS activity.

## Methods

### Experimental animals

Twenty-five adult beagle dogs (body weight 8 to 10 kg) were purchased from Nanjing Medical University Animal Laboratory. They were housed in a room under a 12-h light–dark cycle at 20 to 24 °C. Food and water were freely available throughout the experiment. All protocols were approved by the Institutional Animal Care and Use Committee, consistent with the Guide for the Care and Use of Laboratory Animals (National Research Council).

**Fig. 1:**
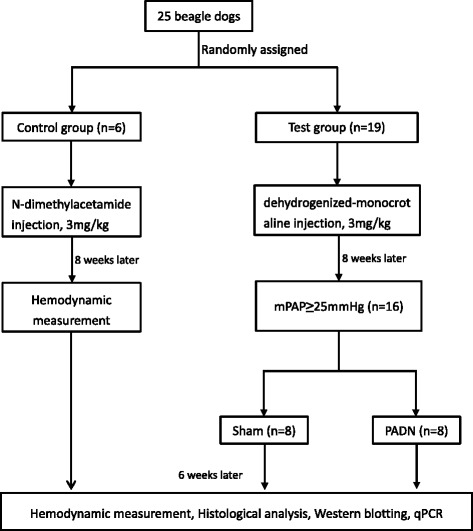
Study flowchat

In the study, each dog was assigned with a computer-generated random number. Then, dogs were allocated into two groups by chance: control group (*n* = 6) and test group (*n* = 19). Dogs in test group were intra-atrially injected with dehydrogenized monocrotaline (DHMCT) at a dose of 3 mg/kg, while the control group received equal amount of N-dimethylacetamide intra-atrial injection. Eight weeks later, beagles in test group with mPAP ≥ 25 mmHg were randomly reassigned into into sham (*n* = 8) and PADN (*n* = 8) groups. PADN catheter connected with a generator was positioned in the PA. Ablation procedure was performed for all animals in the PADN group while the procedure was not performed in the sham group [17]. At the 14^th^ week, all animals were sacrificed when all measurements were completed. Pulmonary and right ventricular tissue was stored stored in a −80 °C refrigerator for further studies (Fig. 1).

### Hemodynamic measurements and PADN Procedure

The methods of hemodynamic measurements and PADN procedure have been described in previous literature [13, 14, 17]. Briefly, after stable anesthesia, a 7-F sheath (Baxter Healthcare Corp.) was inserted percutaneously via the femoral vein and a Swan-Ganz catheter (Edwards Lifesciences, Irvine, California) was positioned at the distal PA for the measurement of mPAP, pulmonary arterial systolic pressure (PASP), pulmonary arterial diastolic pressure (PADP), mean right ventricular pressure (mRVP), right ventricular systolic pressure (RVSP), pulmonary artery occlusion pressure (PAOP) and cardiac output (CO). Then, the PVR [PVR = (mPAP- PAOP) / CO] was calculated. Hemodynamic measurements were tested at three different time points, the beginning of the experiment, the 8^th^ week and the 14^th^ week, separately.

For PADN procedure, a dedicated 7-F catheter (PADNTM; Microport Co., Shanghai, China) was advanced to the main PA along an 8 Fr long sheath, based on pulmonary arteriography. This catheter has a tapered circular tip with 10 pre-mounted electrodes [14]. After the circular tip was released from the sheath, by manipulating a rotating handle, it would be positioned at 3 sites,which were the distal main PA, the conjunction point of the main pulmonary artery trunk lateral wall and ostial left PA, the ostium of left PA, respectively. When the electrodes tightly contacted the endovascular surface, ablation would be performed at 3 sites in turn. The ablation parameters were: a temperature of 45 to 50 °C, energy ≤ 10 W, time 120 s.

### Histological analysis

The samples were excised, fixed in 10 % formalin overnight and then embedded in paraffin [11]. Paraffin-embedded tissue was sectioned into 5 μm thick slices and stained with hematoxylin and eosin (H&E). To evaluate medial wall thickness in muscularized arteries with an external diameter of 80 to 120 μm, we calculated the percentage of medial wall thickness (%MWT), which was [(external diameter- internal diameter)/external diameter] × 100 %. At least ten pulmonary arteries per section were observed and photographed using an Olympus-BHS microscope (San Jose, California) attached to a QImaging Retiga 4000RV digital camera (Surrey, British Columbia, Canada). Two skilled investigators who had no knowledge of the group assignment took photographs and calculating the %MWT, separately.

### Western blots

Lung and heart tissues were lysed with a buffer containing RIPA (Beyotime, China), 10 % phosphatase inhibitor (Roche Applied Science, Germany) and 1 % proteinase inhibitor (Sigma, USA). Bicinchoninic acid assay was used to measure the protein concentration. Equal amounts of the samples were loaded on 10 % SDS–PAGE gels. Then, protein was transferred to a PVDF membrane (Millipore Corporation, USA) and incubated for 1 h at room temperature in blocking solution (5 % non-fat milk). The membrane were incubated overnight at 4 °C in blocking solution containing primary antibodies. Then, it was washed and incubated with horseradish peroxidase conjugated secondary antibody (Cell Signaling Technology, USA) for 1 h at room temperature. After a second wash, the membrane was developed using enhanced chemiluminescence substrate (Millipore Corporation, USA). The band intensities were analyzed using Image J software (National Institutes of Health, Bethesda, USA). Primary antibodies against renin, Ang II, ACE, mineralocorticoid receptor (MR), BNP, phospho-ERK1/2 (Thr202 + Tyr204) and β-actin were purchased from BIOSS Biotechnology (bs-6184R, bs-0587R, bs-0439R, bs-1850R, bs-7132R, bs-3016R, bs-0061R). Primary antibodies against Ang II type 2 receptor (AT2 receptor) and ANP were obtained from Santa Cruz Biotechnology (sc-9040, sc-18811).

### RNA quantitative reverse transcriptase-polymerase chain reaction analysis (qPCR)

Total RNA was extracted using TRIzol reagent (Invitrogen, USA). For mRNA expression analysis, total RNA was reverse transcribed into cDNA using the PrimeScript RT reagent Kit (TakaRa Biotechnology, China), then it was amplified according to the instructions of the SYBR Premix Ex Taq kit (TakaRa Biotechnology, China) on an ABI7500 system (Grand Island, NY, USA). In addition, the equation 2^-ΔΔCt^ was used to determine the relative amount of mRNA in specific target genes. Primer sequences for dog GAPDH are 5’- AGTGGATATTGTCGCCATCA -3’ (forward) and 5’- CAACATACTCAGCACCAGCA -3’ (reverse), for dog AT1 receptor are 5’- ACTGACTTTGCCACTATG -3’ (forward), 5’- ATGATGCAGGTGACTTTT -3’ (reverse).

### Statistical analysis

SPSS 22.0 (IBM Corporation, Armonk, USA) was used for statistical analysis. Data were expressed as mean ± standard deviation (SD). The normality test for all variables was performed using the Shapiro-Wilk test. For normally distributed data, the unpaired Student’s *t*-test was used for binary comparisons, while one-way ANOVA was employed for multiple comparisons. For skewed data distribution, differences in variables were analyzed using the Wilcoxon signed rank or Friedman test. *P* < 0.05 was considered statistically significant.

## Results

### Animal models and mortality

Monocrotaline-induced PH is a reproducible model of progressive pulmonary vasculopathy that reasonably mimics PAH and RV failure [18, 19]. In our study, we established the experimental PAH model by intra-atrially injecting DHMCT at the start point of the research (week 0). Eight weeks later, the successful establishment of PAH model was evaluated by medial wall thickening and the increased mPAP. As a result, sixteen dogs in the test group were successfully established and included in the further study, leaving three dogs excluded. At the 14^th^ week, two dogs in the sham group and one dog in the PADN group died, leaving six dogs in sham group and seven dogs in the PADN group.

### Hemodynamics and RV function after PADN

**Fig. 2:**
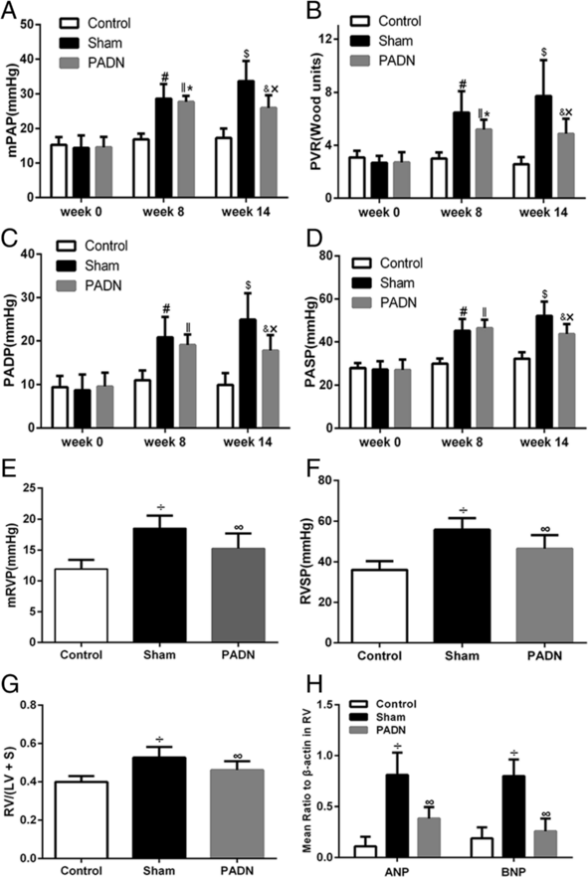
Hemodynamic parameters and RV function in three groups. PADN reversed the development of PAH, demonstrated by the decrease of mPAP (**a**), PVR (**b**), PADP (**c**) and PASP (**d**). Meanwhile, PADN improved RV function, demonstrated by reduced mRVP (**e**), RVSP (**f**), RV/(LV + S) (**g**), ANP and BNP (**h**). # *P <* 0.05 compared with the control group in week 8. ‖*P <* 0.05 compared with the control group in week 8. $ *P <* 0.05 compared with the control group in week 14. & *P <* 0.05 compared with the control group in week 14. * *P <* 0.05 compared with the PADN group in week 14. ×*P <* 0.05 compared with the sham group in week 14. ÷ *P <* 0.05 compared to the control group. ∞P *<* 0.05 compared to the sham group

The hemodynamic data of three time points (week 0, week 8 and week 14) among the three groups were shown in Table 1. There was no significant difference in the baseline hemodynamic parameters among the three groups. At the 8^th^ week, an elevation of mPAP was observed in test group compared with control group (28.64 ± 4.22 mmHg vs 16.87 ± 1.66 mmHg, *P <* 0.05), as well as increased PADP, PASP and PVR in test group, which revealed that the PAH model was successfully established. In the PADN group, mPAP, PVR, PADP and PASP were significantly reduced compared with sham group (Table 1, Fig. 2a to d). However, heart rate and PAOP did not show significant changes among the three groups. Taken together, we believed that PADN can relieve monocrotaline-induced PAH.

**Table 1:** Changes in the hemodynamic parameters in three groups

	Control (*n* = 6)	Sham (*n* = 6)	PADN (*n* = 7)
Heart beats/min			
Week 0 Week 8 Week 14	147.71 ± 25.09 138.57 ± 22.87 134.43 ± 37.95	160.00 ± 17.06 157.70 ± 22.26 159.60 ± 23.81	158.67 ± 13.37 165.89 ± 10.75 159.67 ± 10.14
PVR, Wood units			
Week 0 Week 8 Week 14	3.08 ± 0.50 2.99 ± 0.46 2.57 ± 0.54	2.68 ± 0.52 6.47 ± 1.61^*^ 7.72 ± 2.69^*^	2.70 ± 0.78 5.18 ± 0.75^**,***^ 4.87 ± 1.13^**,****^
mPAP, mmHg			
Week 0 Week 8 Week 14	15.29 ± 2.24 16.87 ± 1.66 17.27 ± 2.77	14.40 ± 3.57 28.64 ± 4.22^*^ 33.72 ± 5.76^*^	14.56 ± 3.00 27.71 ± 1.72^**,***^ 25.94 ± 3.67^**,****^
PADP, mmHg			
Week 0 Week 8 Week 14	9.41 ± 2.62 11.02 ± 2.24 9.93 ± 2.71	8.70 ± 3.62 20.84 ± 4.72^*^ 24.90 ± 6.10^*^	9.56 ± 3.21 19.10 ± 2.37^**^ 17.79 ± 3.58^**,****^
PASP, mmHg			
Week 0 Week 8 Week 14	27.96 ± 2.24 29.98 ± 2.29 32.24 ± 3.02	27.30 ± 3.80 45.22 ± 5.48^*^ 52.14 ± 6.63^*^	27.11 ± 4.70 46.40 ± 3.93^**^ 43.70 ± 4.58^**,****^
mRVP, mmHg			
Week 0 Week 8 Week 14	10.49 ± 1.54 11.10 ± 1.68 11.93 ± 1.48	9.80 ± 3.19 14.96 ± 2.61^*^ 18.52 ± 2.07^*^	10.11 ± 2.20 14.89 ± 1.83^**^ 15.25 ± 2.45^***,****^
RVSP, mmHg			
Week 0 Week 8 Week 14	32.01 ± 3.69 33.38 ± 3.35 36.06 ± 4.30	32.40 ± 7.03 49.06 ± 6.76^*^ 55.84 ± 5.75^*^	31.89 ± 6.45 50.00 ± 4.18^**^ 46.40 ± 6.77^**,****^
PAOP, mmHg			
Week 0 Week 8 Week 14	4.57 ± 1.40 5.29 ± 2.14 6.00 ± 1.63	4.70 ± 1.64 6.20 ± 2.25 4.90 ± 2.02	4.89 ± 1.69 5.67 ± 2.83 5.89 ± 2.09
CO, L/min			
Week 0 Week 8 Week 14	3.39 ± 0.35 3.86 ± 0.84 4.40 ± 1.24	3.61 ± 0.84 3.55 ± 0.66 4.02 ± 1.10	3.69 ± 0.85 4.22 ± 0.77 4.18 ± 0.63

Values are mean ± SD. ^*^ *P <* 0.05 compared with the control group. ^**^ *P <* 0.05 compared with the control group. ^***^ *P <* 0.05 compared with value at week 14. ^****^ *P <* 0.05 compared with the sham group

*PVR* pulmonary vessel resistance, *mPAP* mean pulmonary arterial pressure, *PADP* pulmonary arterial diastolic pressure, *PASP* pulmonary arterial systolic pressure, *mRVP* mean right ventricular pressure, *RVSP* right ventricular systolic pressure, *PAOP* pulmonary artery occlusion pressure, *CO* cardiac output

The RV function was evaluated from three aspects. First, in hemodynamics, despite CO among the three groups didn’t show significant difference, the mRVP and the RVSP increased in sham group compared with control group. After PADN, these values decreased compared to sham group (Table 1, Fig. 2e and f). Then, RV/(LV + S), a hallmark of RV function, was calculated and found to increase in dogs with PAH while reduce after PADN operation (Fig. 2g). Thirdly, as markers of myocardial stress, the levels of ANP and BNP are correlated with myocardial dysfunction and BNP provides prognostic information for PAH diagnosis and follow-up assessments [1]. Thus, levels of ANP and BNP in right ventricles (RV) of the dogs were tested in the study. As presented in Fig. 2h, the levels of ANP and BNP in the right ventricular tissue were higher in sham group with PAH induction than in control group, representing RV dysfunction caused by PAH. However, the levels of ANP and BNP were decreased in dogs performed with PADN, which indicate that PADN can ameliorate the RV function in dogs with PAH.

These results above showed that the PADN procedure led to improvements in hemodynamics and RV function in an experimental PAH model.

### PA remodeling

**Fig. 3:**
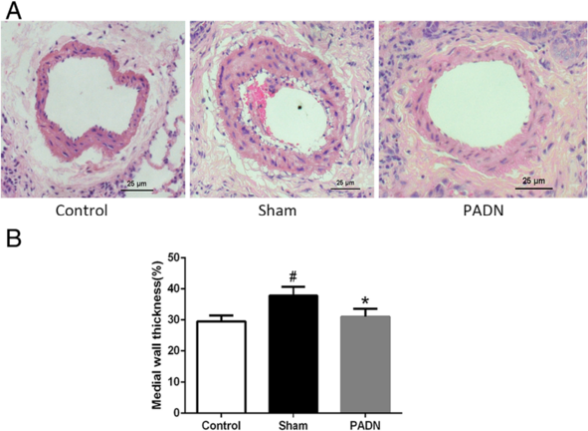
PA remodeling. PADN ameliorated pulmonary arterial remodeling. **a** Representative morphologic images of pulmonary arterial structure in different groups. Sections were stained with hematoxylin and eosin (×200). **b** Bar diagram showed the difference of the %MWT in different groups . #*P <* 0.05 compared with the control group. *P < 0.05 compared to the sham group

Figure 3a showed the representative pictures of hematoxylin and eosin–stained lung sections obtained from dogs in three groups. Pulmonary vessel thickening and luminal stenosis owing to muscularization were observed in the sham group compared with the control and PADN groups. The %MWT, a marker of pulmonary arterial remodeling, was also calculated (Fig. 3b). In the sham group, the %MWT increased (sham group, 37.85 ± 2.80 % vs control group, 29.54 ± 1.85 %; *P* < 0.05). After PADN, it was 33.04 ± 4.41 %, significantly lower than that in the sham group. These data demonstrated that PADN could ameliorate pulmonary vascular remodeling.

### Effects of PADN on the RAAS activity in lung tissue

**Fig. 4:**
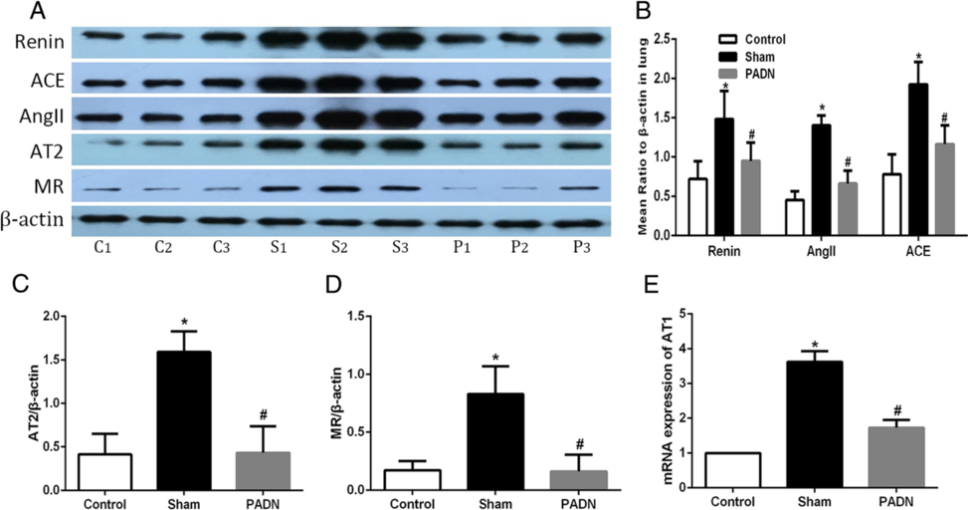
Influence of PADN on the pulmonary RAAS activity. PADN inhibited the local RAAS activity in lung tissue. **a** Representative western blot images of renin, ACE, AngII, AT2, MR and β-actin in pulmonary tissue. **b**–**d** Bar diagram showed intensity data of western blot images, all data were normalized by β-actin. **e** Bar diagram showed data of mRNA expression of AT1 receptor in three groups. C1, C2, C3 : contol group; S1, S2, S3 : sham group; P1, P2, P3 : PADN group. *P < 0.05 compared to the control group. #*P <* 0.05 compared to the sham group

Main components of the RAAS in lung tissue, namely, renin, ACE, Ang II, AT2 receptor and MR, were tested by Western blotting. Real-time PCR was used in detecting AT1 receptor messenger RNA (mRNA). DHMCT-injection was characterized by overexpression of renin, ACE, Ang II, AT2 receptor and MR. PCR results showed a more than threefold increase of AT1 receptor mRNA in lung sections in the DHMCT-injected dogs as compared to the dogs from the control group. PADN treatment in dogs significantly decreased the expression of the mentioned proteins observed in sham group as well as the transcription of AT1 receptor in the PADN group. The results implied that PADN could partially reverse the DHMCT-induced RAAS overexpression in lung tissue (Fig. 4).

### Effects of PADN on the RAAS activity in the right heart

**Fig. 5:**
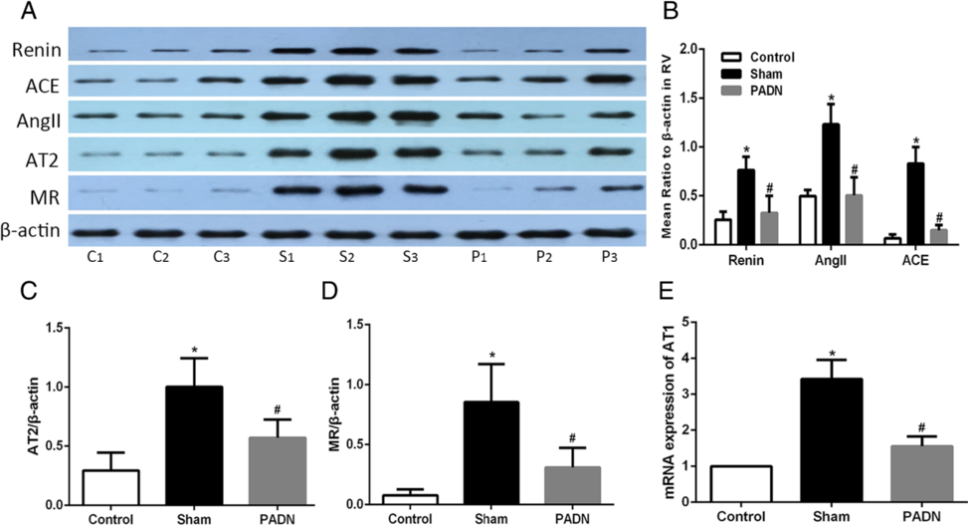
Influence of PADN on the cardiac RAAS activity. PADN reversed RAAS expression in the right heart. **a** Representative western blot images of renin, ACE, AngII, AT2, MR and β-actin in right heart. **b**–**d** Bar diagram showed intensity data of western blot images, all data were normalized by β-actin. **e** Bar diagram showed data of mRNA expression of AT1 receptor in different groups. C1, C2, C3 : contol group; S1, S2, S3 : sham group; P1, P2, P3 : PADN group. *P < 0.05 compared to the control group. #*P <* 0.05 compared to the sham group

In the study, we also tested the transcription and expression of RAAS in the right heart tissue of the above three groups. Injection of DHMCT in dogs resulted in an obvious increase in the transcription and expression of RAAS, which paralleled with the results in lung tissue. In contrast, the transcription and expression went down for PADN group (Fig. 5). The results indicate that PADN can inhibit the local RAAS in cardiac tissue.

### Effects of PADN on signaling of the AT1 receptor

**Fig. 6:**
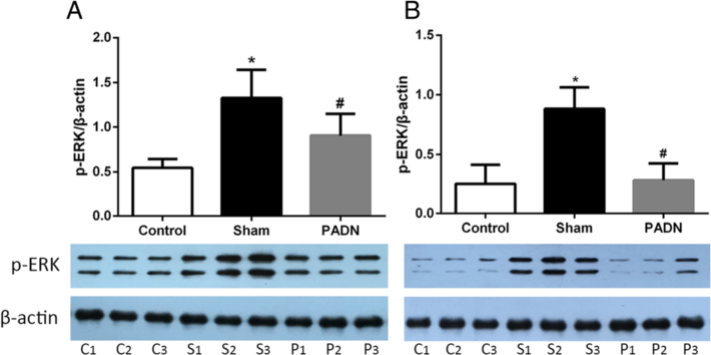
Influence of PADN on the ERK1/2 activity in lung and right heart. ERK1/2 activity was inhibited after PADN. Western blotting analysis for p-ERK1/2 in pulmonary tissue (**a**) and right ventricular tissue (**b**). p-ERK1/2 : phosphorylated form of ERK1/2, C1, C2, C3 : contol group; S1, S2, S3 : sham group; P1, P2, P3 : PADN group. *P < 0.05 compared to the control group. #*P <* 0.05 compared to the sham group

As mentioned above, the mRNA of AT1 receptor was observed to decrease in dogs with RV dysfunction after PADN operation. To test whether the observed decrease in AT1 receptor resulted in decreased receptor signaling, we dectected the ERK1/2 activity, one downstream target of AT1 receptor [20, 21], also an important regulator of cell proliferation. As shown in Fig. 6, ERK1/2 activity (phosphorylated ERK1/2) was significantly increased in dogs with DHMCT injection (sham group). Phosphorylated ERK1/2 was obviously reduced in PADN group in comparison with sham group both in pulmonay (Fig. 6a) and right ventricular tissue (Fig. 6b). Thus, we found protein levels of phosphorylated ERK1/2 showed the same results as the mRNA of AT1 receptor. All these findings together suggest that the AT1 receptor expression and signaling are decreased in dogs with PAH after PADN.

## Discusion

Our study not only explored the impact of PADN on pulmonary vascular remodeling and RV dysfunction, but also investigated the change of RAAS activity after PADN. We provided evidence: a) that PADN attenuated the PAH induced by DHMCT treatment in the beagles, as evidenced by the absence of a significant increase in mPAP and pulmonary vascular wall thickness, b) that PADN treatment improved RV function marked by reduction of the ANP, BNP and RV/(LV + S) elevation caused by DHMCT injection, and c) the local RAAS activity both in lung and right heart was activated in the pathogenesis of PAH, while it was inactivated after PADN operation. Thus, our results suggest that downregulation of the local RAAS activity may be associated with the improvement of abnormal hemodynamics and RV dysfunction by PADN.

As is widely known, the primary function of the RAAS is to regulate intravascular volume to maintain blood pressure [22]. The prevailing evidence suggests that RAAS activity is mainly controlled by the renin, which cleaves angiotensinogen to Ang I. Ang I is hydrolyzed to Ang II by ACE. Then, Ang II, an important role in the development of PAH [23], binds to both the AT1 and AT2 receptors. AngII binds to the AT1 receptor to promote the growth of pulmnary artery smooth muscle cells and stimulate aldosterone synthesis. Aldosterone increases vascular endothelial and smooth muscle cell oxidant stress, which has been implicated in the pathogenesis of PAH [22]. Patients with PAH often have a low cardiac output, to compensate, the RAAS is up-regulated in the pathogenesis of PAH and RV hypertrophy [23–26]. A lot of drugs, such as renin inhibitors, ACE inhibitors and AT1 receptor antagonists, aiming at interfering with the RAAS signaling have been put into wide clinical administration for PAH treatment [27]. Besides the experimental studies, some clinical studies have also revealed similar phenomenon of up-regulation in local pulmonary RAAS activity for patients with iPAH [12, 28], which implies that activated RAAS may be a common mechanism of PAH sharing in mammals.

PADN is a new treatment for PAH based on the operation of SNS [13]. The therapeutic effects of PADN on PAH in patients [13] and experimental models [14, 17] have been reported and accepted. However, for existing experimental and clinical researches, little has been done to explore the influence of PADN operation on local RAAS, as well as its corresponding connection with PAH and RV dysfunction.

Since the RAAS is modulated by the sympathetic adrenergic nervous system, it is now well accepted that the SNS has a close relationship with the RAAS activity. Evidence has revealed the role for the SNS in the modulation of RAAS in producing and maintaining renovascular hypertension [29]. It has been found in patients with PAH, systemic RAAS activity is upregulated, based on findings of upregulated SNS activity [15]. Since PADN induces severe SN injury and abolishes PAH [14], we hypothesized that the SN injury induced by PADN may influence the local RAAS activity and therefore act protectively to pathogenesis of PAH and RV dysfunction.

In our study, we did find a significant increase in renin, Ang II and ACE, which was the same for AT1 receptor, AT2 receptor and MR expression in the pulmonary and right ventricular tissue, acompanied with thickening of the vascular wall and altering of hemodynamic parameters indicating PAH and RV dysfunction. We also tested one downstream tyrosine kinases of the AT1 receptor, phosphorylated ERK1/2. It was observed that phosphorylation of ERK1/2 was increased both in pulmonary and cardiac tissues of dogs with PAH. Consistent with previous investigations, our results showed that local RAAS activity was activated in PAH and RV dysfunction.

In accordance with previous investigations [13, 17], our study showed that the PADN treatment effectively suppressesed PA medial wall thickening and improved hemodynamics. What’s more, PADN improved right ventricular function, which was marked with a significant decrease of ANP, BNP and RV/(LV + S). Meanwhile, PA wall thickness in dogs with PAH was significantly reversed, which could explain the reduction in RV afterload and improvement of RV performance by PADN. Futhermore, to explore the role of RAAS both in the pathogenesis of PAH and after PADN procedure, we also evaluated the local RAAS activity in dogs after PADN. As a result, a significant decrease in the main components of local RAAS in both lung and right heart was observed, implying that the decrease of local RAAS activity may have a relationship with PADN operation. Moreover, p-ERK1/2 expression, which was a downstream mediator of AT1 receptor, was also decreased after PADN. Taken together, these data above suggest that PADN ameliorate s pulmonary arterial remodeling and improves right ventricular function. What’s more, the phenomenon can be related to the inhibited local RAAS activity in pulmonary and cardiac tissue. The effect may be based on the SN injury by PADN procedure.

However, there are some limitations in our study. First, several methods are available to establish a PH model in living animals [30]. Whether regulation of local RAAS is effective in other animal models is an interesting question for future investigation. Next, the mechanism for PADN to treat PAH is complex. Although our work showed that local RAAS was inhibited after PADN, the results could not exclude the involvements of other molecules participating in regulating the RAAS activity either directly or indirectly. Finally, the change of RAAS activity is a continuous process after PADN, but we only measured local RAAS activity in dogs at the time point of 6 weeks after PADN. In brief, further experiments should be done to record the dynamic changes of both the local RAAS and some other associated indicators to reveal the complex mechanisms in the process after PADN.

## Conclusions

Our results demonstrate that PADN treatment attenuates thickening of pulmonary vessels and improves RV function, and this effect is associated with the downregulation of of the local RAAS activity. Hopefully, our study would help better understand the mechanisms of improvements of PA remodeling and cardiac function reversing by PADN.

## Abbreviations

%MWT: The percentage of medial wall thickness
DHMCT: Dehydrogenized-monocrotaline
mPAP: Mean pulmonary arterial pressure
PA: Pulmonary artery
PADN: Pulmonary artery denervation
PAH: Pulmonary arterial hypertension
RAAS: Renin-angiotensin-aldosterone system
RSD: Renal sympathetic denervation
RV: Right ventricle/ ventricular
SN: Sympathetic nerve
SNS: Sympathetic nervous system
